# Neuronal Correlates of Auditory Streaming in Monkey Auditory Cortex for Tone Sequences without Spectral Differences

**DOI:** 10.3389/fnint.2018.00004

**Published:** 2018-01-30

**Authors:** Stanislava Knyazeva, Elena Selezneva, Alexander Gorkin, Nikolaos C. Aggelopoulos, Michael Brosch

**Affiliations:** ^1^Speziallabor Primatenneurobiologie, Leibniz-Institute für Neurobiologie, Magdeburg, Germany; ^2^Laboratory of Psychophysiology, Institute of Psychology, Moscow, Russia; ^3^Center for Behavioral Brain Sciences, Otto-von-Guericke-University, Magdeburg, Germany

**Keywords:** stream segregation, build up, complex tone, phase spectrum, auditory cortex, monkeys, cocktail party effect, neuronal firing

## Abstract

This study finds a neuronal correlate of auditory perceptual streaming in the primary auditory cortex for sequences of tone complexes that have the same amplitude spectrum but a different phase spectrum. Our finding is based on microelectrode recordings of multiunit activity from 270 cortical sites in three awake macaque monkeys. The monkeys were presented with repeated sequences of a tone triplet that consisted of an A tone, a B tone, another A tone and then a pause. The A and B tones were composed of unresolved harmonics formed by adding the harmonics in cosine phase, in alternating phase, or in random phase. A previous psychophysical study on humans revealed that when the A and B tones are similar, humans integrate them into a single auditory stream; when the A and B tones are dissimilar, humans segregate them into separate auditory streams. We found that the similarity of neuronal rate responses to the triplets was highest when all A and B tones had cosine phase. Similarity was intermediate when the A tones had cosine phase and the B tones had alternating phase. Similarity was lowest when the A tones had cosine phase and the B tones had random phase. The present study corroborates and extends previous reports, showing similar correspondences between neuronal activity in the primary auditory cortex and auditory streaming of sound sequences. It also is consistent with Fishman’s population separation model of auditory streaming.

## Introduction

When humans are presented with a periodically recurring sequence of two alternating sounds, they report hearing either a single auditory stream that consists of both sounds or two independent auditory streams each of which consists of only one of the sounds. The perceptual grouping, or auditory streaming, depends on the tempo with which the sounds are presented and on their similarity in feature space. This has been shown for sounds differing in frequency (van Noorden, 1975), amplitude modulation rate (Grimault et al., 2002), spectral composition (Singh and Bregman, 1997; Vliegen and Oxenham, 1999; Cusack and Roberts, 2000; Roberts et al., 2002; Dolležal et al., 2012), or location (Hartmann and Johnson, 1991). For some sequences, there is a build-up of auditory stream segregation, i.e., it takes several seconds until subjects perceive two streams (Bregman, 1978; Anstis and Saida, 1985; Roberts et al., 2002; but see Carlyon et al., 2001; Cusack et al., 2004; Denham and Winkler, 2006; Deike et al., 2012).

Behavioral studies suggest that other mammals (Moss and Surlykke, 2001; Izumi, 2002; Ma et al., 2010; Selezneva et al., 2012; Christison-Lagay and Cohen, 2014) as well as nonmammalian animals (Hulse et al., 1997; Fay, 1998, 2000; MacDougall-Shackleton et al., 1998; Farris and Ryan, 2011; Itatani and Klump, 2014) can also perceptually group sound sequences in ways similar to how humans group them. This implies that different species share neuronal mechanisms underlying perceptual grouping. To the best of our knowledge, build-up of auditory stream segregation has not yet been shown in psychophysical studies on animals.

Several studies have unraveled brain mechanisms that may explain auditory streaming on the level of the neuronal firing. In a seminal study, Fishman et al. (2001, 2004) reported that the same individual neurons responded to both sounds when a sequence of two alternating sounds is perceived as a single auditory stream, but responded predominantly to only one of the two sounds when expected to be perceived as two separate auditory streams. Generally, the similarity of the neuronal responses to the two alternating sounds decreased as the difference in stimulus frequency increased. Based on this observation, Fishman et al. (2004, 2012) proposed that a neuronal correlate of the perception of two separate auditory streams is the presence of two groups of activated neurons and that the perception of a single auditory stream is based on a single group of activated neurons. For ambiguous sound sequences, which are perceived either as one or two streams, there is partial overlap of the activated neurons. This neuronal correlate was described in the primary auditory cortex in the two cited studies and in other studies for tones differing only in frequency (Kanwal et al., 2003; Scholes et al., 2015) or only in source location (Middlebrooks and Bremen, 2013; Yao et al., 2015). It has also been identified in the auditory brainstem (Pressnitzer et al., 2008). Correlates of auditory streaming have also been found in nonmammalian animals (Bee and Klump, 2004, 2005; Schul and Sheridan, 2006; Itatani and Klump, 2011, 2014). Further evidence for an involvement of the auditory system in auditory streaming has been obtained by Micheyl and colleagues (Micheyl et al., 2005; Pressnitzer et al., 2008; Bee et al., 2010), who reported that neuronal responses change during the presentation of an alternating tone sequence in a way that is similar to the psychophysical build-up of auditory stream segregation in humans.

Here, we investigated, in macaque monkeys, neuronal correlates of perceptual grouping of sequences composed of complex sounds, which have so far been studied only in birds (Itatani and Klump, 2011). We closely followed the psychoacoustical study of Roberts et al. (2002), who tested sequences of tone complexes that were composed of identical harmonics. They found that humans hear either one or two auditory streams, depending on the similarity of the phase spectra of the tone complexes. These experiments demonstrated that, for these and other sound sequences, perceptual grouping must result from neuronal mechanisms other than those based on the degree of overlap of the activated neurons in tonotopically organized neuronal maps (Fishman et al., 2004) or on “peripheral channeling” (Hartmann and Johnson, 1991). To find neuronal correlates of perceptual grouping and build up of auditory stream segregation for such sound sequences in the auditory cortex, we recorded and analyzed neuronal activity of awake macaque monkeys while they were passively exposed to repeating triplets of tone complexes that differed only in phase spectrum. Our study also provides information about neuronal bases of the discrimination and identification of such tone complexes.

## Materials and Methods

### Subjects

The data in the present report were obtained from three awake adult monkeys (*Macaca fascicularis*), two males and one female. The animals had participated in previous studies involving behavioral tasks and microelectrode recordings from their auditory cortices. Each monkey was fitted with a head holder for restraint and a recording chamber (21 mm diameter) positioned in the right (monkeys E and M) or left (monkey W) temporal regions of the skull and centered on Horsley-Clarke coordinates A6.4 to A8.0 and D19 to D21.2. Details of the surgery are given elsewhere (Brosch and Scheich, 2008; Mylius et al., 2015). During the experiment, each monkey was awake (with wake state controlled by monitoring the local field potentials and video of the monkey) and squatted quietly in a primate chair with its head fixated via the head holder. The recordings were conducted in a double-walled, soundproof isolation booth (IAC 1202-A). The experiments were carried out in accordance with and approved by the animal care and ethics authority of the state of Saxony-Anhalt (Landesverwaltungsamt Halle).

### Auditory Stimuli

**Figure 1:**
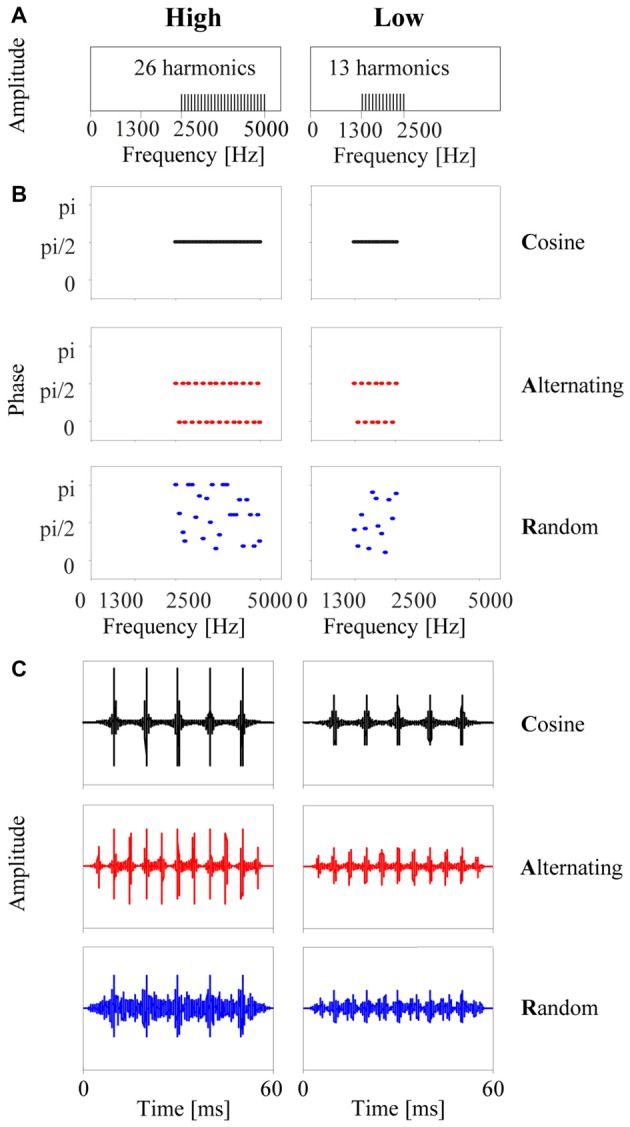
The six types of tone complexes used in the present study. **(A)** Each stimulus consisted of only upper harmonics of 100 Hz, either in a high passband from 2500 Hz to 5000 Hz (left column) or in a low passband from 1300 to 2500 Hz (right column). **(B)** Phase spectra: for each passband, three types of tone complexes were generated by adding the harmonics either in cosine phase (C-tones), in alternating phase (A-tones, with odd harmonics in cosine phase and even harmonics in sine phase), or in random phase (R-tones). **(C)** The resulting time courses of the three tone complexes for each passband. Note that the C-tone and R-tone have five amplitude maxima separated by 10 ms, which corresponds to an envelope modulation frequency of 100 Hz. The A-tone has eleven amplitude maxima separated by 5 ms, which corresponds to an envelope modulation of 200 Hz.

The stimuli were tone complexes with a (missing) fundamental frequency of 100 Hz and designed to have identical spectra, closely resembling the stimuli used by Roberts et al. (2002) in their human psychophysical study. Each stimulus was composed of 26 or 13 unresolved harmonic components in either a high (2500–5000 Hz) or a low (1300–2500 Hz) passband, respectively (Figure 1A). All frequency components were equal in amplitude. Three types of tone complexes were generated by adding components in cosine phase (C-tones), alternating phase (A-tones, with odd harmonics in cosine phase and even harmonics in sine phase), or random phase (R-tones, with phases in the range of 0 to π). Each tone was 60 ms in duration, including 10-ms onset and offset ramps. Figure 1B shows the time courses of the three tone complexes in the high and low passband. Modulation filtering revealed that the dominant frequency of the envelope modulation was 100 Hz in the C-tones and R-tones and 200 Hz in the A-tones.

The tone complexes were arranged in triplets, and each triplet consisted of either three C-tones (CCC), a C-tone followed by an A-tone and another C-tone (CAC), or a C-tone followed by an R-tone and another C-tone (CRC). The triplets were repeated 75 times to form a stimulus sequence. A different phase randomization was used for each of the 75 R-tones in the CRC sequence, but the same CRC sequence was presented on each experimental day. The tones were presented with either a fast or a slow repetition rate. For the fast repetition rate, the parameters matched those used by Roberts et al. (2002) to study auditory streaming of such sequences in humans. The first and second tones in a triplet were each followed by 40 ms of silence, and the third tone was followed by 140 ms of silence. Hence, sequences could also be considered to consist of an alternation between two tones at a rate of 10 Hz (1/100 ms), but with every fourth tone replaced by silence. For the slow repetition rate with an alternation rate of 2.5 Hz, the first and second tones in the triplet were each followed by 340 ms of silence, and the third tone was followed by 740 ms of silence. The slow repetition rate was used to assess whether and how neurons in the auditory cortex responded to each of the three tone complexes and to estimate poststimulatory effects on the neuronal responses to the tone complexes presented at the fast repetition rate. Twelve sequences were used in total and differed in phase spectrum (C, A, R), passband (low, high) and repetition rate (slow, fast) of the tones (the audio files are available here^1^).

The auditory stimuli were generated using MATLAB (Version 2007b), DA converted, amplified (Pioneer A-202), and presented to both ears through wideband in-ear earphones (Etymotic Research ER-4S). To assess the best frequency (the frequency of the tone that elicited that largest number of action potentials) and response latency (the first time bin after tone onset with a significantly elevated number of action potentials) of the auditory cortical neurons, we presented a random sequence of 400 pure tones at 40 different frequencies and 70 dB SPL (for further details of the stimulation and data analysis, see Brosch et al., 1999).

### Neurophysiology

Recordings of multiunit activity from the auditory cortex were performed with a five-electrode system (Thomas Recording). Microelectrodes were oriented at an angle of ~45° in the dorsoventral plane such that they penetrated the dura at approximately a right angle and reached the auditory cortex in the lower bank of the lateral sulcus after traversing the parietal cortex. The signals from each electrode were amplified and bandpass filtered (0.5–5 kHz). The action potentials of several neurons near the electrode tip were detected using threshold crossing and spike duration; their time stamps were stored in a data acquisition system (Cheetah, Neuralynx). Based on the spatial distribution of best frequencies of the multiunits at different sites, we inferred that most recordings were in the primary auditory cortex.

### Data Analysis

For each multiunit and each of the 12 sequences of tone triplets, we computed a peristimulus time histogram (PSTH) with a bin size of 1 ms to determine how the firing rate varied relative to the onset of a triplet. We used all 75 triplets for this analysis to be in correspondence with the results presented in Figure 3 of the psychophysical study of Roberts et al. (2002). We characterized the stimulus encoding by each multiunit using three response measures based on the PSTH in the time window from 9 ms to 100 ms after the onset of each tone in the triplet. (1) The first measure was the rate response, the mean firing rate in the 91-ms time window. The response of a multiunit was considered excitatory if the mean firing rate was significantly higher than the undriven rate and inhibitory if the mean firing rate was significantly lower than the undriven rate (Wilcoxon signed rank test, *p* < 0.05). The undriven rate was estimated from a 91-ms period starting after the silent interval following the third tone of the triplet (1209–1300 ms after the beginning of the triplet). (2) The second response measure was the (temporal) response pattern, which consists of the number of spikes in each of the 91 bins of the PSTH. (3) The third measure described the phase locking of the firing to the dominant envelope modulation frequencies of the tone complexes, either 100 Hz or 200 Hz. To this end, we computed the amplitude spectrum from the response pattern. The amplitude at either 100 Hz or 200 Hz was used as a measure of the phase-locked response. We used a bootstrap analysis to determine whether the phase-locked response was statistically significant, i.e., whether the amplitude was greater, at a *p-level* < 0.05, than the corresponding average amplitude obtained from surrogate PSTHs (*n* = 10,000, computed from simulated spike trains with the same mean firing rate as the original spike train but with randomly distributed interspike intervals; see also Brosch et al., 2015).

To assess whether the neuronal firing of a multiunit reflected the way in which different triplets are perceptually grouped when stimuli are presented at a fast repetition rate (Roberts et al., 2002; redrawn in Figure 6A), we adapted the approach originally used by Fishman et al. (2004). They quantified the similarity between neuronal responses to the tones in the sequence by calculating the ratio of the response to a non-best-frequency tone and the response to a best-frequency tone. They found that the more frequently sequences were perceived as a single auditory stream, the higher the response ratio or the similarity. Guided by these observations, we hypothesized that, for sequences of tone complexes with different phase spectra, the degree of similarity of the neuronal responses to the three tones of the triplet also reflects how the tones are perceptually grouped. Because the incidence of humans reporting one auditory stream is highest for CCC triplets, intermediate for CAC triplets, and lowest for CRC triplets, we expected the similarity of the neuronal responses to be: (1) higher for CCC triplets than for CAC triplets and to be (2) higher for CAC triplets than for CRC triplets. (3) Because CRC triplets composed of tones in the low passband are more often perceived as one auditory stream than those composed of tones in the high passband, the similarity of the responses should be higher for the former than for the latter. (4) Because CCC triplets in both high and low passbands are always perceived as one auditory stream, the neuronal responses should be similar. (5) Because humans usually perceive CRC triplets as consisting of one auditory stream with rapidly repeated C-tones and another auditory stream with slowly repeated R-tones, the responses to the first and second C-tone should be more similar to each other than to the responses to the first C-tone and the R-tone.

The response ratio cannot be used to quantify, with a single value, the response similarity for sequences composed of three tones instead of two tones. Therefore, to quantify the response similarity for rate responses and phase-locked responses, we instead used the reciprocal of the standard deviation of the responses to the three tone complexes in a triplet. The standard deviation is given by the square root of the mean squared difference between the responses to each tone complex (*r*_1_, *r*_2_, *r*_3_) and the mean of the three responses (*m*):

$${\left(\frac{{\left(r_{1}-m\right)}^{2}\;+\;{\left(r_{2}-m\right)}^{2}\;+\;{\left(r_{3}-m\right)}^{2}}{3}\right)}^{1/2}$$

The similarity of the response patterns was quantified by the pairwise Pearson correlation coefficient. The coefficients were calculated for pairs C vs. A, C vs. R and A vs. R. Compared PSTHs were obtained by averaging the 75 responses to the second tone complex in each triplet (CCC, CAC and CRC). An additional coefficient was calculated for the pair C vs. C by comparing PSTHs from responses to the third tone complex in triplets CAC and CRC.

To test whether neuronal activity in the primary auditory cortex was related to the build-up of auditory stream segregation in humans, we adapted the neurometric approach introduced by Micheyl and colleagues for sequences of pure tones (Micheyl et al., 2005; Pressnitzer et al., 2008; Bee et al., 2010). We thus used signal detection theory to determine whether the neuronal responses evoked by the second and third tone in a particular triplet provide information that an ideal observer can use to decide whether one or two auditory streams are present. This was done for the two tone sequences having the most extreme dynamics of auditory streaming during the 30-s presentation interval (Roberts et al., 2002). The first was the CRC sequence in the high passband whose perception changed slowly from 1 to 2 auditory streams during a large portion of the presentation interval (Roberts et al., 2002). This sequence corresponds best to the pure tone sequences having a frequency separation of three semitones used by Micheyl and coworkers (see Figure 4 in Micheyl et al., 2005). The second was the CCC sequence in the high passband that humans almost always perceive as one auditory stream (Roberts et al., 2002). This sequence corresponds best to the pure tone sequences having a frequency separation of one semitone used by Micheyl et al. (2005).

We divided each of the tone sequences into six 5-s-long non-overlapping time segments (as in Figure 4 of Roberts et al., 2002). For a particular time segment and sequence, we counted the number of spikes occurring during each presentation of the second and third tone in the triplet. To decide whether one or two auditory streams were present, we compared the means of the two spike-count distributions to different spike-count thresholds. When only one mean exceeded the threshold, the probability of hearing two streams was equal to 1. When both means exceeded the threshold, the probability of hearing two streams was defined as the ratio of the distance of the first mean from the threshold and the distance of the second mean from the threshold. For each time segment, 101 thresholds were tested. One set of thresholds was generated for each multiunit by determining the minimum and maximum spike counts observed in any time segment of the CRC and CCC sequences and dividing this range into 100 equally spaced intervals. For each threshold, a neurometric “build-up” curve was produced to show the probability of hearing two streams within each of the six time segments. We selected the threshold yielding the smallest sum of squared differences between the psychophysical (described by Roberts et al., 2002) and neurometric curves for CRC and CCC sequences.

## Results

**Figure 2:**
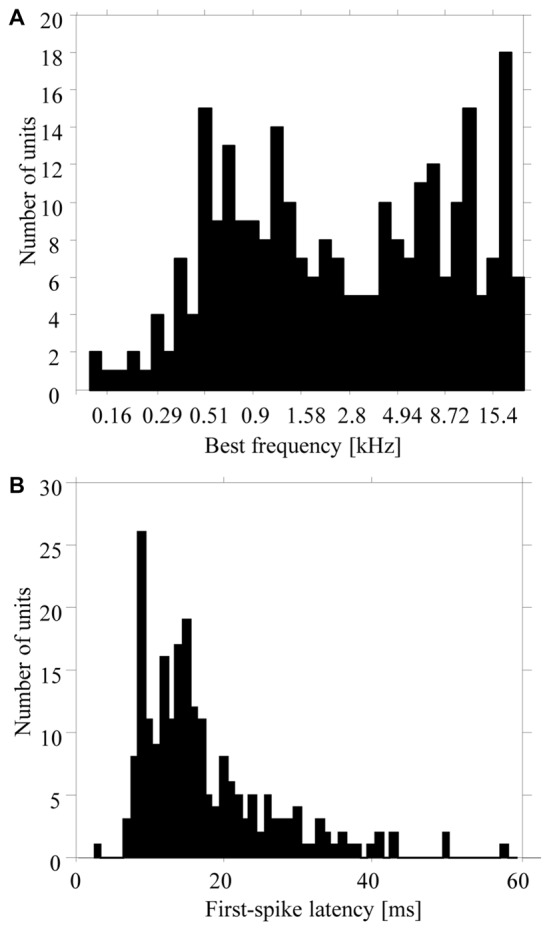
Distribution of best frequencies **(A)** and first-spike latencies **(B)** of the 270 multiunits in the auditory cortex used in the present study. Values were obtained from responses to pure tones having different frequencies.

Our results are based on multiunit activity recorded from 270 sites in the primary auditory cortex of three monkeys (120 sites in monkey M, 92 in monkey E, and 58 in monkey W). Additional recordings from 112 sites were not used because their multiunit activities were not stable during the 740-ms period of silence between the six tone sequences presented at the slow repetition rate. The best frequencies of the 270 multiunits ranged from 0.1 kHz to 23 kHz (Figure 2A). All multiunits showed excitatory or inhibitory responses to pure tones with frequencies from 1300 Hz to 5000 Hz, i.e., to the frequency range that was used for the tone complexes in the low and high passband (e.g., Figure 4B). First spike latencies ranged from 9 ms to 80 ms, with a median of 14 ms (Figure 2B).

**Figure 3:**
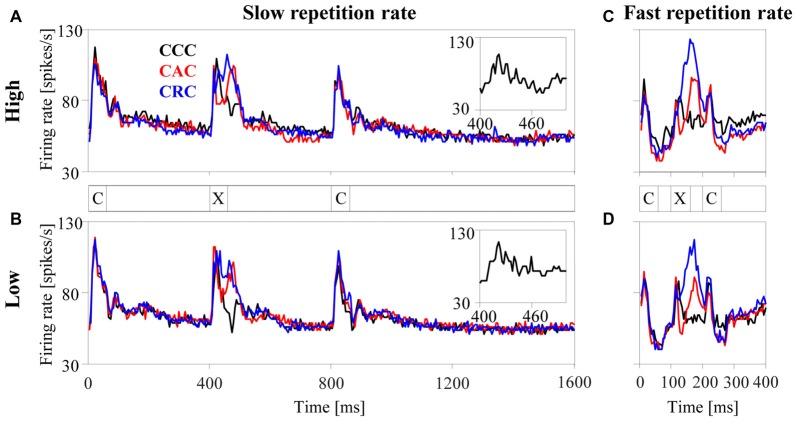
Population responses of 270 multiunits in the auditory cortex to harmonic tone complexes. The tone complexes were arranged in triplets (marked by the letters C and X) and presented sequentially at either a slow (2.5 Hz, panels **A,B**) or a fast (10 Hz, panels **C,D**) repetition rate. Triplets were composed of three C-tones (CCC triplet, black curve), of a C-tone, an A-tone and a C-tone (CAC triplet, red curve), or of a C-tone, an R-tone and a C-tone (CRC triplet, blue curve). All tone complexes consisted of harmonics in either the high passband (panels **A,C**) or the low passband (panels **B,D**) and had a duration of 60 ms. For display purposes, the bin width was set to 5 ms. The insets replot the population responses to the second C-tone with a smaller bin width of 2 ms to better show the phase locking to the envelope modulation frequency at 100 Hz.

**Figure 4:**
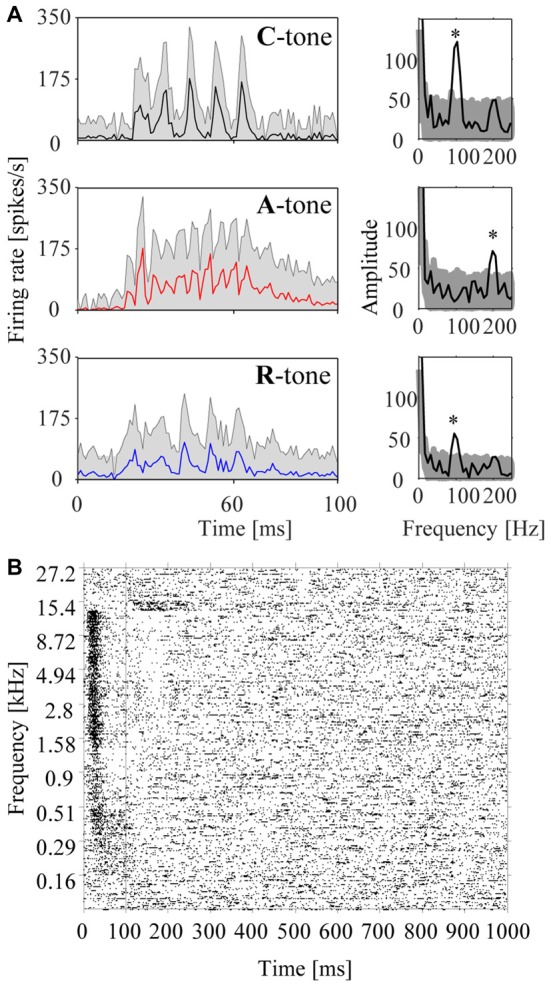
Responses of a representative multiunit in the auditory cortex to harmonic tone complexes and pure tones. **(A)** Responses to harmonic tone complexes. The left column shows the peristimulus time histograms (PSTHs) for C-tones (top row), A-tones (middle row), and R-tones (bottom row) in the low passband. Note that the firing rate varies with the envelope modulation frequency of the tone complex (i.e., the firing rate peaked five times during the presentation of the C-tones and R-tones, and eleven times during the presentation of the A-tones (see Figure 1B). The shaded area reflects the standard errors of the mean firing rate. The periodicities of the firing are also reflected in the amplitude spectra computed from the histograms (right column). The stars indicate frequency bins with a significant increase in amplitude (see “Materials and Methods” section). The shaded area reflects the 95% range of 10,000 bootstraps. **(B)** Frequency response area of the multiunit. The raster plot shows the firing in response to 400 pure tones of 40 different frequencies (each repeated 10 times) that were randomly presented during 100 ms with 900 ms salient interval.

### Tone Complexes with Different Phase Spectra Evoke Different Neuronal Firing in the Primary Auditory Cortex

Here, we first describe how neurons in the auditory cortex respond to tone complexes that have the same amplitude but a different phase spectrum (Figure 1). In addition to providing details about the neuronal underpinnings of the discrimination and identification of tone complexes differing in their phase spectra, this section serves as a basis for the interpretation of the main results regarding the neuronal underpinnings of auditory streaming for sequences composed of such tone complexes.

The left panels in Figure 3 show the average response of the 270 multiunits in the auditory cortex to each tone complex when presented with the slow repetition rate of 2.5 Hz. For tone complexes in either the high (panel A) or the low (panel B) passband, the firing rate sharply increased ~10 ms after the tone onset and subsequently returned within ~100 ms to the undriven firing rate when no tones were present (i.e., from 1209 ms to 1300 ms after triplet onset). A comparison of the responses to the second tone in each triplet (the only position where the tone complex can differ, marked by X in Figure 3) revealed that the population firing depended on the phase spectrum of the tone complex. Firing rates were generally highest for R-tones (blue curve), intermediate for A-tones (red curve), and lowest for C-tones (black curve). These differences resulted mostly from differences in the firing rate between ~35–100 ms after tone onset. During this interval, the A-tones and R-tones elicited higher firing rates than the C-tones. By contrast, the initial portions of the responses to the three tone complexes were quite similar. The (temporal) response pattern of the 270 multiunits therefore differed between the tone complexes, implying that the response pattern of neurons contains information about the phase spectra. The population responses were also phase locked to the envelope modulation frequency of the tone complexes, particularly to that of the C-tones (see insets). This implies that the spike timing carried information about the dominant envelope modulation frequency of the tone complexes.

In addition to the population responses, we also analyzed individual multiunits to characterize the sensitivity of the auditory cortex to tone complexes having different phase spectra. To this end, we computed PSTHs from the neuronal responses to the individual tones in the triplets. Figure 4A shows the PSTHs of a representative multiunit for C-tones, A-tones and R-tones. The interval from 9 ms to 100 ms after tone onset was used to calculate the rate response, the response pattern, and the phase-locked responses.

**Figure 5:**
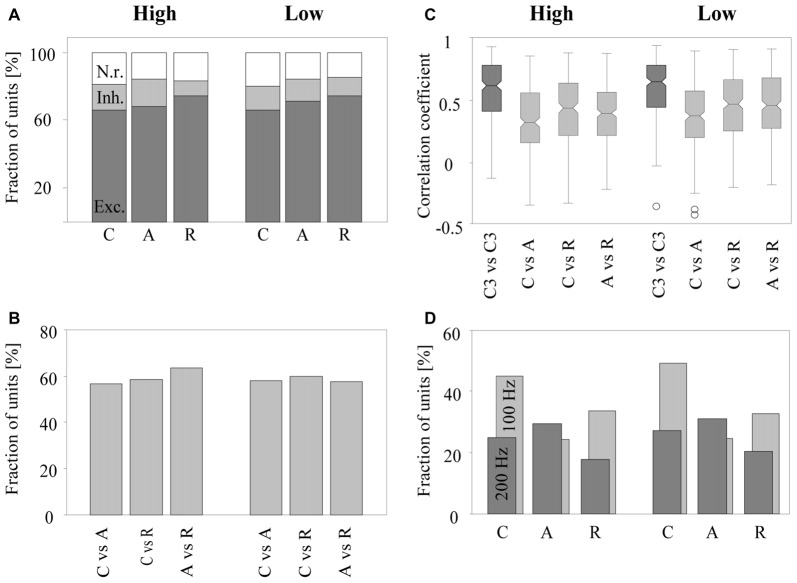
Sensitivity of neurons in the auditory cortex to the phase spectrum of harmonic tone complexes. Results are based on 270 multiunits. **(A)** Fraction of multiunits with excitatory (dark gray bar), inhibitory (light gray bar), or no (white bar) rate responses to C-tones, A-tones, and R-tones with components in the high (three left bars) or in the low (three right bars) frequency passband. **(B)** Fraction of multiunits whose rate responses differed significantly between a C-tone and an A-tone, a C-tone and an R-tone, and an A-tone and an R-tone in the two passbands. **(C)** Distribution of correlation coefficients for response patterns in the two passbands. The dark gray boxes show correlation coefficients computed from the response patterns of two C-tones in the third position but from the triplet CAC and the triplet CCC (C3 vs. C3). The light gray boxes show correlation coefficients computed from the response patterns of two different tones in the second position in the CCC, CAC, and CRC triplets (C vs. A, C vs. R and A vs. R). The bottoms and tops of each notched box are the 25th and 75th percentiles of the samples, respectively. The line in the middle of each box is the sample median. The whiskers extend to the most extreme data points not considered outliers (circles). **(D)** Fraction of multiunits with significant phase-locked responses to C-tones, A-tones and R-tones in the two passbands. Phase locking at 100 Hz is indicated by light gray bars, and phase locking at 200 Hz is indicated by dark gray bars.

The rate response was defined as the mean firing rate in the time window from 9 to 100 ms after tone onset. It was deemed excitatory if it was significantly higher and inhibitory if it was significantly lower than the undriven firing rate (Wilcoxon signed rank test, *p* < 0.05). We found that the majority of the 270 multiunits (~2/3) exhibited an excitatory rate response to the tone complexes. Smaller fractions of the multiunits exhibited an inhibitory rate response (~1/7) or no rate response (~1/6). Figure 5A shows, for both low and high passbands, the percentage of multiunits exhibiting each type of rate response when the C-tones, A-tones and R-tones were in the second position in the triplets. In 63.7% of the multiunits, the rate response depended significantly on the phase spectrum of the tones (Friedman test, *p* < 0.05), with no quantitative difference between the two passbands. Wilcoxon signed rank tests between pairs of tone complexes (*p* < 0.05, Bonferroni corrected) revealed that similar percentages of multiunits (56.7%–63.3%) discriminated C-tones from R-tones, C-tones from A-tones, and A-tones from R-tones (Figure 5B). On average, the response rates were highest for R-tones, intermediate for A-tones, and lowest for C-tones. In the high passband, three multiunits did not exhibit responses to the C-tones, four multiunits to the A-tones and three multiunits to the R-tones. In the low passband, nine multiunits did not respond to the C-tones, three to the A-tone and twelve to the R-tones.

The similarity of the response patterns to different tone complexes was quantified by the Pearson correlation of the firing in the interval from 9 ms to 100 ms after onset of the second tone. This was done for all possible pairwise comparisons: C-tones vs. A-tones, C-tones vs. R-tones and A-tones vs. R-tones. The median correlation coefficients of the 270 multiunits were in the range of 0.33 and 0.49 for the six comparisons that could be performed between pairs of different tone complexes in the high and low passbands (Figure 5C). These correlation coefficients were significantly smaller than those for the response patterns of two C-tones presented in the third position but from different triplets (e.g., the third tone in a CAC triplet vs. the third tone in a CCC triplet, signed rank test, *p* < 0.0001). The latter correlation coefficients had medians in the range of 0.64 and 0.67, indicating that response patterns varied considerably over time.

To assess phase locking, we computed the amplitude spectrum from the PSTH and used a bootstrap procedure to identify firing components that are significantly synchronized with the envelope modulation frequency of 100 Hz or 200 Hz (*p* < 0.05; see “Materials and Methods” section). A representative multiunit showing strong phase-locked responses to the dominant envelope modulation frequencies of the C-tones, A-tones and R-tones is shown in Figure 4. In our sample, almost two-thirds of the multiunits (63.7%) exhibited significant phase-locked responses to at least one tone complex. Percentages for each tone complex and passband are shown in Figure 5D. These percentages were all greater than the chance level, which was determined by quantifying periodic firing at 100 or 200 Hz in the silent interval during which no tone was presented. Significant phase-locked responses were found most frequently at the dominant envelope modulation frequency of the tone complex, which was 100 Hz for the C-tones and R-tones and 200 Hz for the A-tones. In addition, more multiunits exhibited significant phase-locked responses to the C-tones than to the R-tones, possibly reflecting that the C-tones had a larger proportion of the total power at 100 Hz than the R-tones. Finally, more multiunits had significant phase-locked responses to the C-tones and R-tones than to the A-tones. This might be due in part to the decreasing capability of cortical neurons to phase lock their responses to faster modulations (Schreiner et al., 1997; Joris et al., 2004; Yin et al., 2011; Malone and Schreiner, 2012).

It is unlikely that the neuronal responses described so far would change substantially if the tone complexes with different phase spectra contained silent intervals longer than the 340 ms used in the present study (Figure 3). This statement is based on a comparison of the responses to the first and second C-tones in the triplets, which were preceded by silent intervals of 740 ms and 340 ms, respectively. This comparison revealed that the rate responses of the 270 multiunits decreased slightly and that their response patterns and phase-locked responses did not change systematically. These observations are in line with, and extend, previous observations for pairs of brief stimuli in which poststimulatory effects in the auditory cortex lasted a few 100 ms (Brosch and Schreiner, 1997; Brosch et al., 1998, 1999; Brosch and Scheich, 2008). By contrast, the neuronal responses to the three tone complexes changed substantially when silent intervals <340 ms were used. This is observed for the condition in which the tone complexes were presented at the fast repetition rate of 10 Hz (Figures 3C,D). At this repetition rate, firing rates were generally lower than those at the slow repetition rate of 2.5 Hz (Figures 3A,B). This appeared to be most pronounced for the C-tones and least pronounced for the R-tones. For the latter, the firing rate could even be enhanced compared to the slow repetition rate. We could not perform a detailed analysis of the effects of even shorter silent intervals (140 ms and 40 ms) on the three response measures and it was unclear whether the firing during a given tone was suppressed or increased. The main reason for this was that, because long sequences of repeated tones were presented, there were not only interactions between nearest tones (as in our previous studies) but also between non-adjacent tones.

### Neuronal Correlates of Auditory Streaming in the Primary Auditory Cortex

After establishing that neurons in the auditory cortex are sensitive to the phase spectrum of tone complexes, we investigated whether their firing also reflects the way in which sequences of such tones are perceptually grouped. To this end, we analyzed neuronal firing recorded in response to the different tone triplets presented at the fast repetition rate of 10 Hz (Figures 3C,D). These triplets were similar to the sequences used in the psychophysical study of Roberts et al. (2002). We followed the approach of Fishman et al. (2004), who showed that the more frequently sequences were perceived as a single auditory stream, the higher the similarity of neuronal responses. We calculated the same three response measures from the neuronal firing as in the first section of the Results and tested whether the similarity of the responses to the tones in the triplets varied in the same way as did human perception (Roberts et al., 2002). For rate responses and phase-locked responses, similarity was quantified by the reciprocal of the standard deviation of the responses in the triplet. For response patterns, similarity was quantified by the average pairwise Pearson correlation coefficient computed from the responses to the triplet.

**Figure 6:**
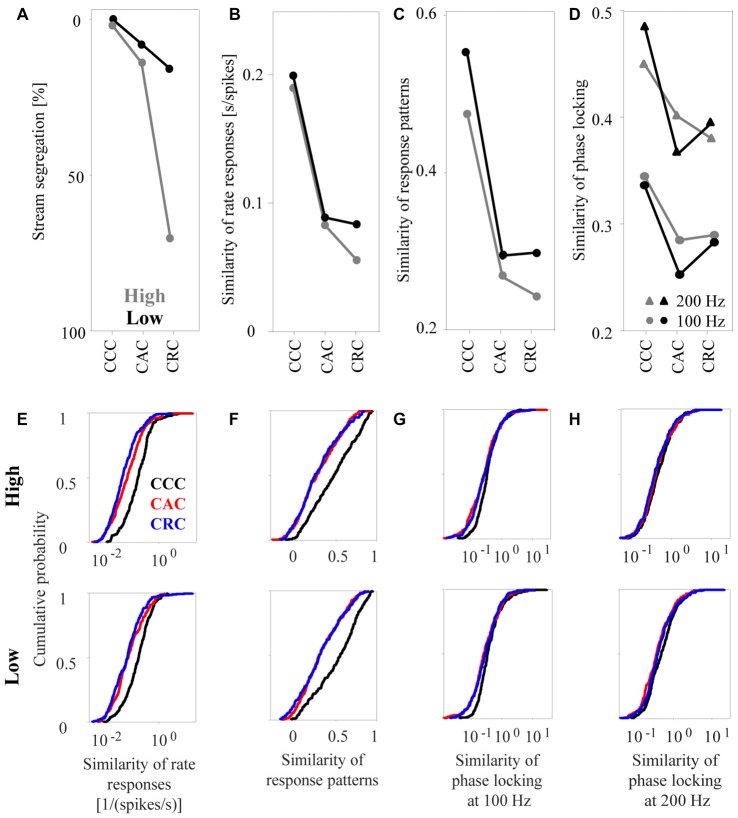
Similarity of neuronal responses in the auditory cortex to sequences of tone complexes. **(A)** Proportion of time during which humans perceived the sequences as segregated (redrawn from Figure 3 in Roberts et al., 2002). Sequences consisted of CCC, CAC, or CRC triplets. The gray and black dots indicate results for tone complexes in the high and low passbands, respectively. **(B)** Median similarity of the rate responses of 270 multiunits for the three triplets in the high passband and the low passband. **(C)** Median similarity of the response patterns. **(D)** Median similarity of the phase-locked responses to 100 Hz (dots) and to 200 Hz (triangles). Standard errors are omitted from the figure to avoid cluttering the main results. The lower panels show cumulative distributions of the similarity of the rate responses **(E)**, of the similarity of the response patterns** (F)**, and of the similarity of the phase-locked responses to 100 Hz **(G)** and to 200 Hz **(H)** for the three tone complexes in the high and low passbands.

From a visual inspection of how the psychophysical measure and the three neuronal response measures depend on the composition of the tone triplets, we find that rate responses corresponded best to the way in which humans perceptually group the triplets (Figures 6A,B). Consistent with the observation that, in humans, the incidence of reporting one auditory stream was highest for CCC triplets, intermediate for CAC triplets, and lowest for CRC triplets (Figure 6A), we found that the median similarity of rate responses of the 270 multiunits was highest for CCC triplets, intermediate for CAC triplets, and lowest for CRC triplets, regardless of whether the tones were in the high (gray curve in Figures 6B,E, upper panel) or low (black curve, Figure 6E, lower panel) passbands. Moreover, consistent with the observation that triplets in the low passband are more frequently perceived as one auditory stream than triplets in the high passband, we found that rate responses of triplets in the low passband had a higher median similarity than rate responses of triplets in the high passband. The relationship between perceptual organization and response similarity was also present in the response patterns (Figures 6C,F). However, a decrease of the similarity of response patterns from CCC triplets to CAC triplets to CRC triplets was only present in the high frequency passband. Unlike human reports, the median similarity of the phase-locked responses of the 270 multiunits did not differ, or even increased, between CAC and CRC triplets in the low passband. The lowest correspondence between the perceptual organization and response similarity was found for the phase-locked responses at both 100 Hz and at 200 Hz (Figures 6D,G,H).

To determine quantitatively whether perceptual organization depends on the similarity of the different response measures, we computed the statistical significance of the differences in response similarity for selected pairs of tone triplets. From the psychophysical observations (see Figure 3 in Roberts et al., 2002), we found that the frequency of reporting “stream segregation” differs between: (1) CCC triplets and CAC triplets and (2) CAC triplets and CRC triplets in the high passband, as well as between (3) CRC triplets in the high and low passbands. (4) By contrast, there is no difference between CCC triplets in the high and low passbands. When we performed pairwise signed rank tests to determine whether the relationships also apply to the response similarity of the 270 multiunits (*p* < 0.05), we found that these four comparisons yielded results compatible with the perceptual data. For response patterns and phase-locked responses, maximally two of the four comparisons yielded significant results.

Finally, we tested the fifth hypothesis, namely that the responses to tones within the same auditory stream are more similar than the responses to tones within different auditory streams. To this end, we analyzed the response similarity of the 270 multiunits during the presentation of CRC triplets in the high passband, which humans usually perceive as two concurrent auditory streams, one consisting of rapidly repeated C-tones and the other consisting of slowly repeated R-tones. The median similarity of the rate responses to the two C-tones (at the 2nd and the 3nd position of the CCC triplet) was significantly higher than the response similarity of the R-tone and the C-tones (signed rank test, *p* < 0.0001).

### Neuronal Correlates of Build-up Auditory Stream Segregation in the Primary Auditory Cortex

**Figure 7:**
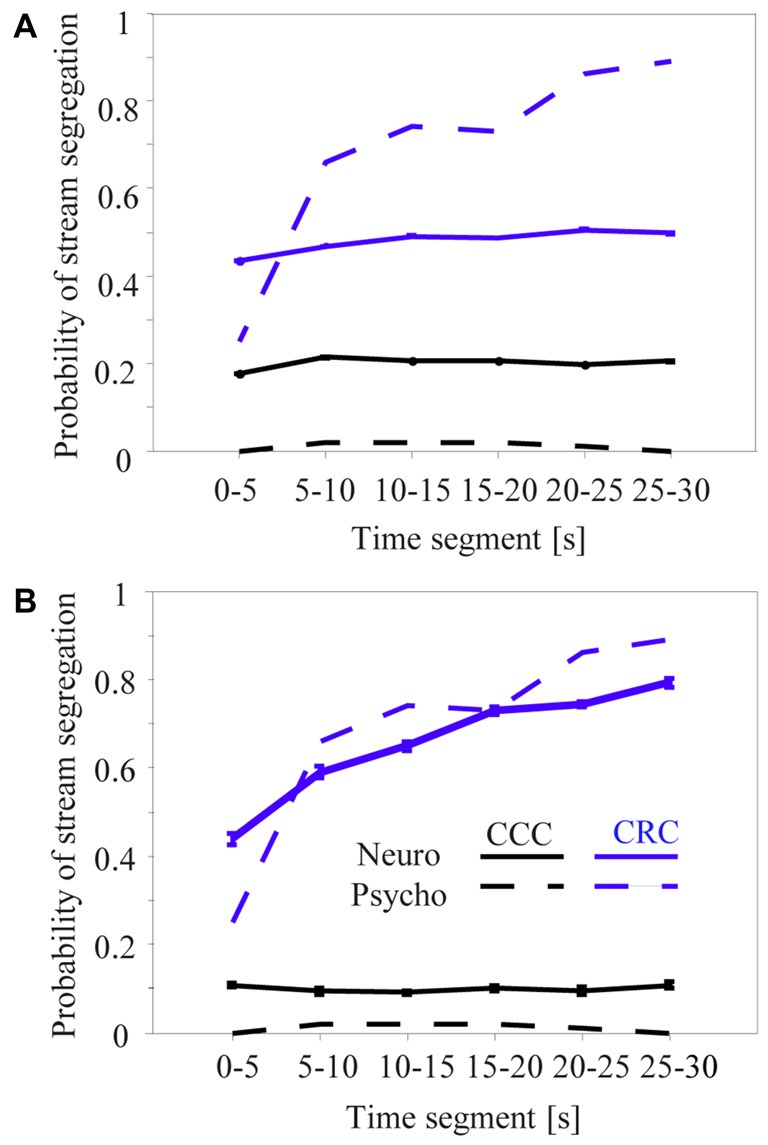
Proportion of time during which sequences of tone complexes in the high passband were heard or classified as two segregated streams across successive 5-s segments. The dashed curves are redrawn from the left panel of Figure 4 in Roberts et al. (2002) and show how the CRC (gray) and CCC (black) sequences were perceived by six human subjects. The solid lines show results of a time-resolved neurometric analysis using signal detection theory to determine whether the neuronal responses evoked by the second and third tone in a triplet provide information that an ideal observer could use to decide if one or two auditory streams is present (for details, see “Materials and Methods” section). Panel **(A)** shows the mean neurometric results of 218 multiunits whose responses differed between the C-tones and R-tones. Panel **(B)** shows the mean neurometric results of the 10 multiunits having the smallest difference between the psychometric and neurometric curves for CRC and CCC sequences. Whiskers on the solid curves of the panels **(A,B)** show standard errors of the means.

Roberts et al. (2002) reported that the perceptual organization of CRC sequences in the high passband was not constant during their presentation time of 30 s. The frequency of perceiving two streams steadily increased from about 27% during the initial 5 s to about 90% after 25 s (Figure 7A, dashed gray curve). By contrast, no such build-up was observed for CCC sequences, which subjects continuously perceived as one stream (Figure 7A, dashed black curve). Because neuronal adaptation was in the order of 2–4 s and thus much faster than psychophysical build-up, we adapted the neurometric approach first used by Micheyl and colleagues to establish relationships between the build-up and the neuronal activity in the primary auditory cortex (Micheyl et al., 2005; Pressnitzer et al., 2008; Bee et al., 2010).

Figure 7 shows the results of the neurometric analysis for different subsamples of the 270 multiunits in the auditory cortex. Our first approach was to include only the 218 multiunits that showed a significant difference between the responses to the C-tones and R-tones in the triplet (signed rank test, *p* < 0.05). This revealed little correspondence between the perceptual build-up curves and the neurometric build-up curves (Figure 7A). In contrast to the perceptual reports obtained for the CRC sequences, the mean neurometric probability of the 2-stream percept remains relatively constant at a value of about 47% during the 30-s presentation time, with a slight increase only, and thus deviates most strongly from the psychometric curve at the beginning and end of the sequence (gray curves). For the CCC sequence, the neurometric analysis yields a relatively constant probability of the 2-stream percept during the 30-s presentation time but is much higher than the psychometric curve (black curves).

We made several unsuccessful attempts to identify a population of neurons whose neuronal build-up curves had a qualitatively better correspondence to psychophysical build-up curves. This included analyzing the entire neuronal population or by applying different physiological selection criteria, such as larger response differences between C-tones and R-tones or higher complexity of frequency response areas of the multiunits. Some individual multiunits in our sample of 270 multiunits had a “good” correspondence between psychophysical and neuronal build-up curves. Figure 7B shows the mean neuronal build-up curve for the ten multiunits with the smallest differences between the psychometric and neurometric curves for CRC and CCC sequences. They showed a clear increase of the 2-stream probability while the CRC sequence was presented, as well as large differences in the 2-stream probability between CCC and CRC sequences.

## Discussion

This is the first study to demonstrate a neuronal correlate of perceptual grouping in the primary auditory cortex for sequences of tone complexes having identical amplitude spectra but different phase spectra. We found that the similarity of neuronal responses corresponds well to the frequencies with which humans perceive sequences of tone complexes to consist of either one or two auditory streams (Roberts et al., 2002). The present study therefore corroborates and extends previous reports also showing correspondences between neuronal activity in the primary auditory cortex and the perceptual grouping of sequences of sounds that differ in frequency (Fishman et al., 2001, 2004, 2017; Kanwal et al., 2003; Noda et al., 2013; Farley and Noreña, 2015; Scholes et al., 2015) or source location (Middlebrooks and Bremen, 2013; Yao et al., 2015).

Correspondences between auditory streaming and neuronal activity in the auditory cortex have also been found in humans for sequences of sounds differing in frequency (Gutschalk et al., 2005; Snyder et al., 2006; Wilson et al., 2007), fundamental frequency (Gutschalk et al., 2007), or source location (Carl and Gutschalk, 2013). For example, Gutschalk et al. (2007) reported that the larger the difference in pitch of the tones in the sequence, the higher the BOLD activation in the auditory cortex of both hemispheres, including the Heschl’s gyrus and planum temporale. In a parallel measurement, the MEG responses to less frequently presented tones were found to increase with differences in fundamental frequency. As in the present study, Gutschalk et al. (2007) also used tone complexes with identical spectral envelopes, but unlike the present study, the tones differed in fundamental frequency.

In contrast to most previous studies, we used sequences composed of tone complexes rather than composed of pure tones. Therefore, we first established that neurons in the auditory cortex are sensitive to the phase relationships between the components of tone complexes. This finding was important for this study because otherwise no differences would have been expected in the neuronal responses to the sequences of such tone complexes. In addition, our study provides further evidence for the view that neuronal activity in the primary auditory cortex contributes to the perceived pitch and roughness or harshness of sounds (Steinschneider et al., 1998). We found that fewer neurons exhibited phase-locked responses to R-tones than to C-tones, consistent with the psychophysical observations in humans that the pitch strength is weakened and the tone complex sounds harsher and more noiselike when the components are summed in random phases (Bilsen, 1973). We also found that neuronal responses are preferentially phase locked to higher frequencies when the components of the tone complex are summed in alternating phase than when the components are summed in the cosine phase (Shackleton and Carlyon, 1994; for nonhuman primates, see Bendor et al., 2012). These results are consistent with earlier observations made for so-called pitch-selective neurons in the low frequency region of the auditory cortex, which also responded differently to unresolved C-tones than to unresolved A-tones (Bendor et al., 2012).

Previous studies have identified, in the auditory forebrain in birds, neuronal correlates of the auditory streaming of sequences of sounds that differ in their phase spectra (Itatani and Klump, 2011; Dolležal et al., 2012). As in our study, these authors also used sounds with C, A and R phase relationships between harmonics to generate sequences of CCC, CAC and CRC triplets. Consistent with our study, they found a correspondence between the rate responses and perceptual grouping in humans (Roberts et al., 2002). In contrast to our study, they also found a correspondence for the phase-locked responses. This disagreement may reflect differences between the auditory systems of birds and mammals or may result from different randomization procedures used to produce the R-tones (which were not specified in the previous studies). We can exclude that the disagreement results from different methods of assessing phase-locked responses, because we found qualitatively similar results when phase locking was quantified by the peak-to-background ratio used by Itatani and Klump (2011).

Virtually all the primary auditory cortex studies cited above found a correlate of auditory streaming in the strength of neuronal activation (Fishman et al., 2001, 2004; Kanwal et al., 2003; Middlebrooks and Bremen, 2013; Noda et al., 2013; Farley and Noreña, 2015; Scholes et al., 2015; Yao et al., 2015). Consistent with these findings, the present study shows a correspondence between auditory streaming and the rate response (the mean firing rate within a fixed interval relative to the tone presentation). We also explored other neuronal firing characteristics and found that both the response patterns and the responses that were phase locked to the dominant envelope modulation frequency had only little correspondence with the auditory streaming of the tone sequences.

Correlates of auditory streaming based on the strength of neural activation were also obtained from the auditory cortex of humans. In MEG measurements (Gutschalk et al., 2005, 2007; Snyder et al., 2006; Carl and Gutschalk, 2013), the amplitude of the M100 component evoked by one of two alternating tones in a sequence increased as a function of frequency separation and repetition rate. Likewise in fMRI studies, BOLD signals in Heschl’s gyrus and other parts of the auditory cortex increased when the sequential tones were made dissimilar and thus perceived more frequently as two streams (Gutschalk et al., 2007; Wilson et al., 2007; Schadwinkel and Gutschalk, 2010). This correlation between neural activity and streaming also exists for ambiguous tone sequences when listeners judged their grouping moment-by-moment (Gutschalk et al., 2005) or when listeners were given instructions that biased them towards integration or segregation (Deike et al., 2012).

Based on recordings at individual sites in the auditory cortex, Fishman et al. (2001) proposed a model for auditory streaming in which the perceptual organization of tone sequences is related to the spatial overlap or separation of the neuronal responses to the tones. Direct experimental support for this model was recently provided by imaging membrane potentials in the auditory cortex using a voltage-sensitive dye (Farley and Noreña, 2015). Our finding that neuronal responses to tone complexes become less similar when the tone complexes differ in phase spectrum is compatible with the model of Fishman et al. (2001). Thus, our findings suggest that the number of neurons responding equally to all tones in the triplets would decrease and the number of neurons responding predominantly to the C-tones or the R-tones would increase for sequences composed of tone complexes. Two neuronal populations would be activated by sequences of tone complexes that have the same amplitude spectrum but different phase spectra and are perceived as two auditory streams, whereas a single neuronal population would be activated by sequences of such tone complexes that are perceived as a single auditory stream. In contrast to the model of Fishman et al. (2004), however, the neuronal populations would not be defined in terms of their frequency sensitivity and thus would not be located at different sites in the tonotopic map of the primary auditory cortex but potentially in other auditory cortical fields. Rather, the populations could differ in their sensitivity to more complex sound features such as pitch and timbre (Schulze et al., 2002; Bendor and Wang, 2005; Bizley et al., 2009; Griffiths and Hall, 2012) or in their neural synchrony (Elhilali et al., 2009).

It is well established that the perceptual organization of a recurring tone sequence changes during its presentation (Anstis and Saida, 1985). The changes are reflected in the so-called build-up of auditory stream segregation which refers to the fact that, for some tone sequences, it takes several seconds until subjects report hearing two auditory streams, particularly if one assumes that subjects initially hear one auditory stream and if results are averaged across trials and subjects (Denham and Winkler, 2006; Deike et al., 2012). For the CRC sequences studied here, Roberts et al. (2002) reported that the tendency for the stimuli to be heard as two streams increased over the course of roughly 20 s. Using an adaptation of the neurometric approach first introduced by Micheyl et al. (2005), we were able to identify a small number of neurons in the auditory cortex whose firing on average changed during the 30-s presentation of the CRC sequences in a way that corresponded well with the average psychophysical build-up behavior described by Roberts et al. (2002). Our results thus corroborate the findings of Micheyl and colleagues who obtained a similar correspondence in the auditory brain stem and auditory cortex for sequences of pure tones (Micheyl et al., 2005; Pressnitzer et al., 2008; Bee et al., 2010). One difference between their studies and our study is the criterion for including neurons in the neurometric analysis. Micheyl et al. (2005) took advantage of the high frequency sensitivity of neurons in the primary auditory cortex and included only neurons for which at least one stimulus tone was at, or very near, the best frequency. In the present study, some sensitivity for the shape of phase spectrum was demonstrated. It is questionable, however, whether this sensitivity is as high as that for pure tones. This might explain why we were unable to identify a physiological parameter to distinguish the minority of neurons with a good correspondence between firing and psychophysical build-up curves from the majority of neurons with no correspondence or a different correspondence. Thus, it is unclear by which mechanism neurons with different neurometric curves can be selected. It is possible that neuronal activity in the primary auditory cortex is less informative about auditory streaming for tone complexes than for pure tones. This calls to consider alternative mechanisms as expressed in the evidence accumulation model by Barniv and Nelken (2015) or in the competition model by Rankin et al. (2015).

Because the monkeys were passively exposed to tone sequences in the present study, it is unknown whether activation patterns in the primary auditory cortex persist and form the basis of perception when the monkeys actively attend to the tone sequences. This issue can be addressed only by performing experiments in which the monkeys’ perceptions are inferred from their behavioral responses. Such experiments have been performed using other tone sequences and have revealed that monkeys perceptually group the sequences in ways similar to how humans group them (Izumi, 2002; Selezneva et al., 2012; Christison-Lagay and Cohen, 2014). A recent study on New World monkeys found that they also perceive the missing fundamental frequency of unresolved harmonic tone complexes and that their pitch perception depends on the phase spectrum (Bendor et al., 2012). In addition, humans and nonhuman primates have largely overlapping audible frequency ranges (Stebbins, 1966) and a similar auditory cortical organization (Hackett, 2007). For these reasons, it is likely that macaque monkeys perceptually group sequences of tone complexes having different phase spectra in a way that is similar to how humans group them (Roberts et al., 2002).

## Author Contributions

SK, ES, AG, NCA and MB (all authors) contributed to design, data acquisition, interpretation, drafting and revising of the work and final approval.

## Conflict of Interest Statement

The authors declare that the research was conducted in the absence of any commercial or financial relationships that could be construed as a potential conflict of interest.

## Footnotes

^1^https://www.researchgate.net/publication/321945864_Sound_files_for_Neuronal_correlates_of_auditory_streaming_in_monkey_auditory_cortex_for_tone_sequences_without_spectral_differences
